# Spatial Cluster Analysis of Fatal Road Accidents From Non-Use of Seat Belts Among Older Drivers

**DOI:** 10.1093/geroni/igaa057.374

**Published:** 2020-12-16

**Authors:** Oluwaseun Adeyemi, Rajib Paul, Ahmed Arif

**Affiliations:** University of North Carolina at Charlotte, Charlotte, North Carolina, United States

## Abstract

Identifying county-level spatial clusters of fatal accidents due to non-use of seatbelt among drivers 65 years and older can help with injury prevention policies and targeted place-based interventions. We estimated the odds and identified hotspots of fatal accidents among drivers 65 years and older (n=57,715) based on a cross-sectional analysis of data from 2010 to 2018 from the Fatality Analysis Reporting System. The outcome variable was fatality status (fatal/non-fatal), and the main independent variable was seatbelt use (not used/used). Other covariates were drunk driving, distracted driving, and speeding while age, gender, and airbag deployment were used as confounders. Rural-urban status of accident location was used as an effect modifier. Odds ratios were calculated from logistic regression. The age-adjusted fatality rate was computed as the crude fatality rate per 100,000 population weighted by the average population composition by age-groups. Spatial autocorrelation was assessed by local Moran’s I, and cluster analysis was performed using the Moran’s I index-derived Z-scores. The median age-adjusted seatbelt-related fatality rate per county was 2.35 per 100,000 population (IQR: 5.60). Not wearing a seatbelt was associated with an 11-fold (Adjusted OR: 11.37; 95% CI: 10.18-12.70) increased odds of a fatal event in metropolitan counties and a 7-fold (Adjusted OR: 7.43; 95% CI: 6.10-9.04) increased odds in rural counties and small towns. Hot spots for seatbelt-related fatal road accidents were found in multiple counties in Texas, South Dakota, and Mississippi. Study findings can be used for county-specific interventions tailored to 65 and older for preventing fatal road accidents.

